# Wedge Resection versus Stereotactic Body Radiation Therapy for Non-Small Cell Lung Cancer Tumors ≤8 mm

**DOI:** 10.3390/curroncol31030116

**Published:** 2024-03-15

**Authors:** Arian Mansur, Zain Saleem, Jorind Beqari, Camille Mathey-Andrews, Alexandra L. Potter, James Cranor, Alexandra T. Nees, Deepti Srinivasan, Margaret E. Yang, Chi-Fu Jeffrey Yang, Hugh G. Auchincloss

**Affiliations:** Division of Thoracic Surgery, Massachusetts General Hospital, Boston, MA 02114, USA; zsale040@uottawa.ca (Z.S.); jbeqari@mgh.harvard.edu (J.B.); camille_mathey-andrews@dfci.harvard.edu (C.M.-A.); apotter8@mgh.harvard.edu (A.L.P.); jcranor@mgh.harvard.edu (J.C.); alexnees003@gmail.com (A.T.N.); dsrinivasan2@mgh.harvard.edu (D.S.); myang18@mgh.harvard.edu (M.E.Y.); cjyang@mgh.harvard.edu (C.-F.J.Y.); hauchincloss@mgh.harvard.edu (H.G.A.)

**Keywords:** lung cancer, NSCLC, stereotactic body radiation therapy, wedge resection, screening

## Abstract

The objective of this study was to evaluate the overall survival of patients with ≤8 mm non-small cell lung cancer (NSCLC) who undergo wedge resection versus stereotactic body radiation therapy (SBRT). Kaplan–Meier analysis, multivariable Cox proportional hazards modeling, and propensity score-matched analysis were performed to evaluate the overall survival of patients with ≤8 mm NSCLC in the National Cancer Database (NCDB) from 2004 to 2017 who underwent wedge resection versus patients who underwent SBRT. The above-mentioned matched analyses were repeated for patients with no comorbidities. Patients who were coded in the NCDB as having undergone radiation because surgery was contraindicated due to patient risk factors (e.g., comorbid conditions, advance age, etc.) and those with a history of prior malignancy were excluded from analysis. Of the 1505 patients who had NSCLC ≤8 mm during the study period, 1339 (89%) patients underwent wedge resection, and 166 (11%) patients underwent SBRT. In the unadjusted analysis, multivariable Cox modeling and propensity score-matched analysis, wedge resection was associated with improved survival when compared to SBRT. These results were consistent in a sensitivity analysis limited to patients with no comorbidities.

## 1. Introduction

There are currently no data from ongoing randomized trials comparing stereotactic body radiation therapy (SBRT) and wedge resection for patients with non-small cell lung cancer (NSCLC) [1,2,3,4]. Additionally, there has been a question of whether SBRT or wedge resection is superior for very small NSCLC tumors given the increasing number of smaller tumors discovered either incidentally or through lung cancer screening over the past decade [5,6,7]. While surgical resection is the preferred treatment option in the National Comprehensive Cancer Network (NCCN) guidelines for patients with T1aN0M0 NSCLC who can tolerate surgery, there is a concern of performing unnecessary surgery for small, potentially benign nodules, and clinicians sometimes opt for watchful waiting while weighing the risks of disease progression [8,9]. This is especially the case for small NSCLC tumors ≤8 mm in size where imaging and fine-needle aspiration biopsy are less conclusive at determining malignancy potential, and the latter may be associated with complications like pneumothorax [8,10,11,12]. However, treatment options like sublobar wedge resection and SBRT are generally well tolerated by most patients and offer attractive alternatives to patients with early-stage NSCLC [13,14,15,16]. Prior work using the Surveillance, Epidemiology, and End Results (SEER) database has suggested a survival benefit for surgery versus non-operative management in patients with NSCLC tumors ≤8 mm [8].

The purpose of this study was to analyze the overall survival of patients with early-stage N0 NSCLC tumors ≤8 mm who undergo wedge resection versus SBRT using the National Cancer Database (NCDB). We hypothesize that patients undergoing wedge resection would have increased survival when compared to those receiving SBRT.

## 2. Methods

### 2.1. Data Source

The NCDB is a nationwide oncology outcomes database that is jointly managed by the American Cancer Society and the Commission on Cancer of the American College of Surgeons. The NCDB captures approximately 72% of all newly diagnosed cases of lung cancer in the United States and Puerto Rico [17,18], and contains over 30 million patient records from over 1500 cancer centers.

### 2.2. Study Design

This study was deemed exempt by the Massachusetts General Hospital Institutional Review Board. The International Classification of Diseases for Oncology, with third edition (ICD-O-3) histology and topography codes, was used to identify all patients diagnosed with ≤8 mm NSCLC in the NCDB between 2004 and 2017. This study period was specifically chosen because information on the Charlson/Deyo comorbidity condition (CDCC) score was available only for cases diagnosed from 2004 onwards and survival data were available for patients diagnosed up to 2017 at the time of analysis.

Only patients who were diagnosed with a single malignancy of ≤8 mm NSCLC and who were treated at the reporting facility were included in the cohort. To minimize selection bias, patients were excluded if they were coded as having received radiation because surgery “was contraindicated due to patient risk factors (comorbid conditions, advance age, etc.)” or if they had a coded history of prior malignancy. Further exclusion criteria included patients who had unknown or missing American Joint Committee on Cancer (AJCC) staging, histology other than adenocarcinoma or squamous cell carcinoma, patients who received induction therapy, and patients who underwent palliative treatment.

### 2.3. Statistical Analysis

Patients with early-stage N0 NSCLC ≤8 mm tumors were grouped based on whether they underwent wedge resection or SBRT. To compare the baseline characteristics and short-term outcomes of our cohort, Student’s *t* tests or Wilcoxon rank sum tests were used for continuous variables and Pearson’s χ^2^ tests or Fisher’s Exact tests were used for discrete variables.

The log-rank test and Kaplan–Meier product limit approach were used to calculate the median survival and 5-year overall survival (OS). Next, a multivariable Cox proportional hazards regression model was used to further analyze the OS of patients by treatment type. The variables that were included in the Cox model were identified to be clinically significant *a priori* and included the following: age, sex, race, income, insurance type, year of diagnosis, treatment facility type, distance between patient’s residence and treatment facility, tumor histology, tumor location, tumor size, and CDCC score.

A propensity score-matched analysis was conducted to match patients by treatment type using similar previously described methods [19]. In summary, propensity scores were constructed and defined as the probability of treatment with wedge surgery versus SBRT, conditional on the same covariates as those used in our Cox models. Next, a radius-matching algorithm with a caliper of 0.01 was used to identify well-matched pairs of patients. After the patients were matched, the balance was assessed using absolute standardized differences, and the OS of the two arms was evaluated using Kaplan–Meier analysis. Secondary outcomes were assessed using Student’s *t* tests or Wilcoxon rank sum tests for continuous variables and Pearson’s χ^2^ tests or Fisher’s exact tests for categorical variables.

To limit selection bias, a sensitivity analysis was conducted in healthier patients with no comorbidities using the above-mentioned analysis: the Kaplan–Meier method and log-rank test, multivariable Cox proportional hazards modeling, and propensity score-matched analysis.

The statistical analyses in this study were performed with Stata/MP software, version 17.0 for Mac (StataCorp, College Station, TX, USA). Significance was determined using a two-sided *p* = 0.05.

## 3. Results

**Figure 1:**
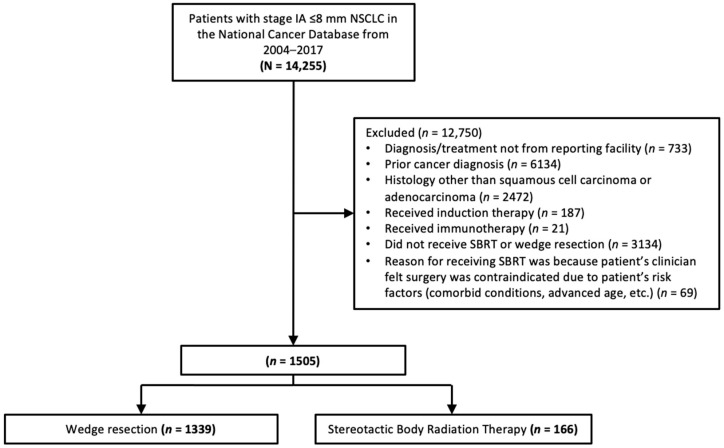
Flow diagram showing schema of study subject selection. NSCLC, non-small cell lung cancer.

The NCDB study cohort consisted of 14,255 patients with stage IA ≤8 mm NSCLC between 2004 and 2017. Of these patients, 1505 (10.56%) met the study inclusion criteria, among which 1339 (89.0%) patients received wedge resection and 166 (11.0%) patients received SBRT (Figure 1).

The baseline demographic and tumor characteristics for these patients are indicated in Table 1. Patients undergoing wedge resection were more likely to be older, non-White and have earlier diagnosis, adenocarcinoma, private insurance, and a higher income.

**Figure 2:**
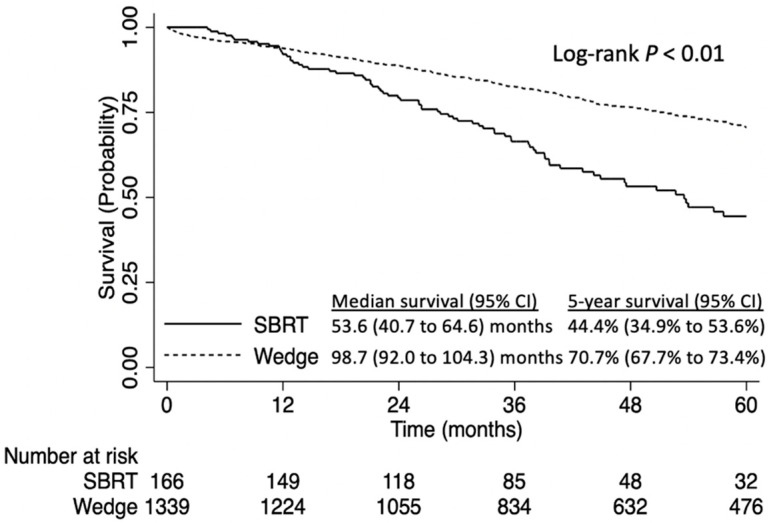
Overall survival of patients with stage IA NSCLC tumors ≤8 mm NSCLC, stratified by stereotactic body radiation therapy (SBRT) versus wedge resection.

Following the Kaplan–Meier analysis, the 5-year OS of patients who underwent wedge resection was significantly greater than those who underwent SBRT (5-year OS: 70.7% [95% CI: 67.7–73.4%] versus 44.4% [95% CI: 34.9–53.6%], log-rank *p* < 0.01, Figure 2).

Wedge resection was significantly associated with improved survival when compared to SBRT (adjusted hazard ratio 0.55 [95% CI: 0.42–0.73], *p* < 0.01, Table 2) in the multivariable analysis.

Propensity score matching was used to create two groups of 130 patients who underwent either wedge resection or SBRT. These groups were well matched by baseline characteristics, and all absolute standardized differences were less than or equal to 15.3 (Table 3), with no significant differences in baseline characteristics.

**Figure 3:**
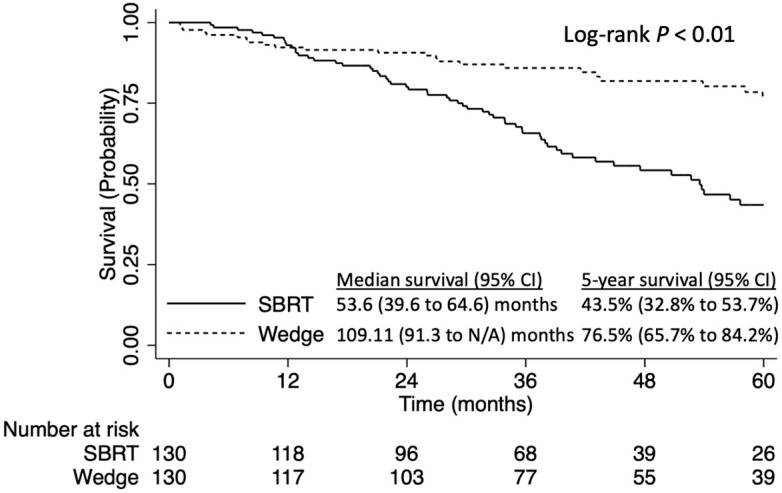
Overall survival of patients with stage IA NSCLC tumors ≤8 mm NSCLC, stratified by stereotactic body radiation therapy (SBRT) vs. wedge resection: propensity score-matched analysis.

Wedge resection was significantly associated with improved survival when compared to SBRT (5-year OS 76.5% [95% CI: 65.7–84.2%] versus 43.5% [95% CI: 32.8–53.7%], log-rank *p* < 0.01, Figure 3).

**Figure 4:**
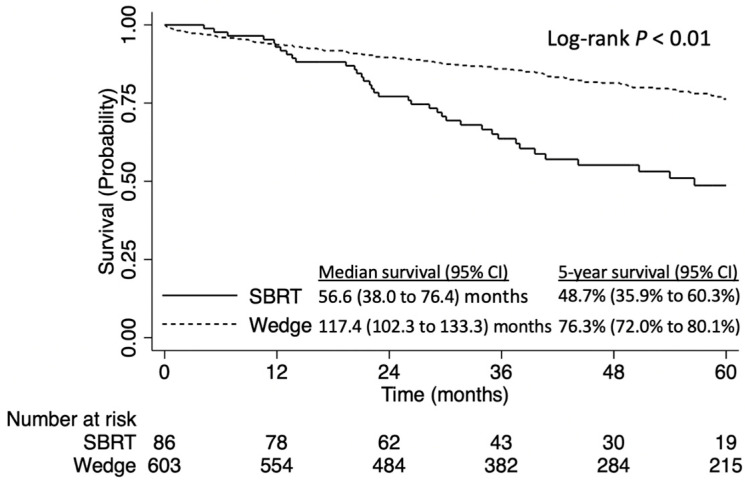
Overall survival of patients with stage IA NSCLC tumors ≤8 mm NSCLC who have no comorbidities, stratified by stereotactic body radiation therapy (SBRT) vs. wedge resection.

In a sensitivity analysis limited to patients with no comorbidities, patients who underwent wedge resection had significantly greater survival when compared to those who underwent SBRT (5-year OS 76.3% [95% CI: 72.0–80.1%] versus 48.7% [95% CI: 35.9–60.3%], log-rank *p* < 0.01, Figure 4).

In the multivariable analysis, wedge resection was significantly associated with improved survival when compared to SBRT (adjusted hazard ratio 0.51 [95% CI: 0.34–0.77], *p* < 0.01, Table 4).

Propensity score-matching was used to create two groups of 65 patients each who underwent wedge resection or SBRT and had no comorbidities. These groups were well-matched based on demographic characteristics, with no significant differences between them and absolute standardized differences less than or equal to 19.6 (Table 5).

**Figure 5:**
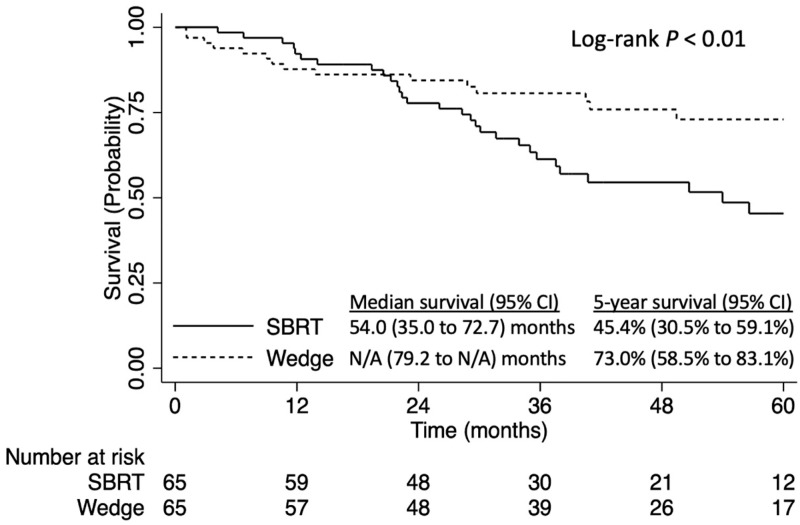
Overall survival of patients with stage IA NSCLC tumors ≤8 mm NSCLC who have no comorbidities, stratified by stereotactic body radiation therapy (SBRT) vs. wedge resection: propensity score-matched analysis.

Wedge resection was associated with better survival than SBRT in the propensity score-matched analysis (5-year OS 73.0% [95% CI: 58.5–83.1%] versus 45.4% [95% CI: 30.5–59.1%], log-rank *p* < 0.01, Figure 5). The findings of this sensitivity analysis were consistent with those of the primary analysis.

**Figure 6:**
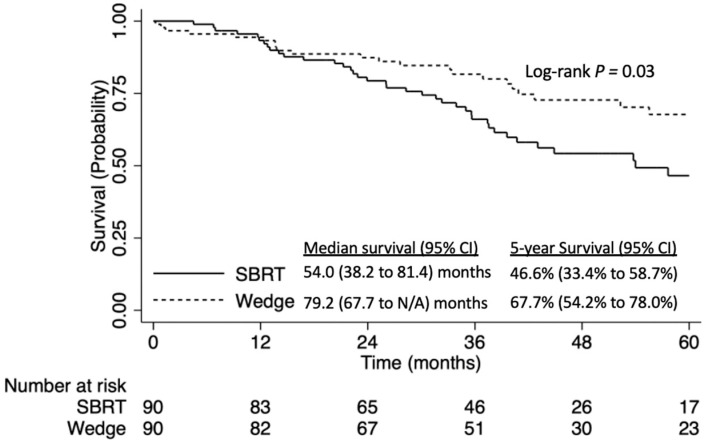
Overall survival of patients with stage IA NSCLC tumors ≤8 mm NSCLC, stratified by ≥50 Gy stereotactic body radiation therapy (SBRT) vs. wedge resection: propensity score-matched analysis.

In another sensitivity analysis where patients in the SBRT arm received a total radiation dose ≥50 Gy, the findings remained consistent with the above-mentioned propensity score-matched analyses (Figure 6) and multivariable analysis (Table 6).

## 4. Discussion

In this study, we evaluated the overall survival of patients who underwent wedge resection versus SBRT for the treatment of patients with early-stage N0 NSCLC tumors ≤8 mm. In both the unadjusted analysis as well as the multivariable Cox modeling and propensity score-matched analysis, wedge resection was associated with a significantly improved 5-year OS when compared with SBRT. In a sensitivity analysis limited to patients who have no comorbidities, both multivariable Cox modeling and propensity score-matched analysis similarly demonstrated that wedge resection was associated with significantly better survival when compared to SBRT. Furthermore, a finding in our analysis was that the SBRT patients had improved survival when compared to the surgery cohort during the first year. While the exact reasons for this cannot be determined from our data, we think this is due to a lower immediate post-treatment morbidity and mortality associated with SBRT when compared to surgery.

To our knowledge, this is the first study to evaluate wedge resection versus SBRT for patients with N0 NSCLC ≤8 mm. Numerous other studies [20,21,22,23,24,25,26,27,28,29,30,31,32,33,34] have compared the outcomes of SBRT with those of wedge resection for early-stage NSCLC but with tumors that are larger and with conflicting results. Our findings have been consistent with the only meta-analysis evaluating wedge resection versus SBRT in clinical stage I NSCLC, which found that wedge resection had a superior OS when compared to SBRT [7]. There are currently three randomized trials comparing SBRT versus surgery [1,2,3,4], but these trials are still ongoing.

A general concern of retrospective studies evaluating SBRT versus surgery for early-stage lung cancer is that unmeasured confounding exists and can lead to biased results [35,36]. In the present study, we tried to reduce selection bias by excluding patients in the SBRT group whose physicians thought that surgery was not recommended or contraindicated due to patient risk factors such as comorbid conditions and elderly age and by conducting a sensitivity analysis limited to patients with no comorbidities.

There are several important limitations to this study. First, it is a retrospective study; therefore, there is a possibility that confounding factors and selection bias exist despite multivariable Cox modeling and propensity score-matched analyses. Specifically, the NCDB does not include key variables such as patients’ smoking status, smoking history, and pulmonary function. Second, detailed patient clinical data such as pulmonary function are not available in the NCDB. We attempted to account for this limitation by including Charlson–Deyo comorbidity scores and performing a sensitivity analysis including healthier patients with no comorbidities. Third, the NCDB does not contain data regarding postoperative complications, disease-free survival, disease-specific survival, and surgeon experience. Finally, the NCDB does not contain radiographic details regarding the tumors, and the consolidation-to-tumor ratios were unavailable; thus, we were unable to differentiate solid tumors from subsolid nodules with mixed solid ground-glass opacity components.

## 5. Conclusions

In this national analysis, patients diagnosed with ≤8 mm NSCLC tumors who underwent wedge resection had improved survival when compared to those who underwent SBRT. Our findings underscore the importance of using a multidisciplinary approach for patients with very small NSCLC tumors, and that operable patients receive comprehensive counseling regarding the differences in survival associated with wedge resection and SBRT. Until prospective, randomized trials are complete, these findings may aid the treatment decision-making process in patients with tumors that are discovered incidentally or through lung cancer screening.

## Figures and Tables

**Table 1:** Baseline characteristics for patients with stage IA NSCLC tumors ≤8 mm NSCLC who received SBRT versus wedge resection.

Patient Characteristic	SBRT (n = 166)	Wedge Resection (n = 1339)	*p* Value
Age (median, IQR), years	71.0 (66.0, 78.0)	68.0 (61.0, 73.0)	<0.01
Sex, n (%)			0.24
Male	70 (42.2%)	502 (37.5%)	
Female	96 (57.8%)	837 (62.5%)	
Race, n (%)			0.04
White	153 (92.2%)	1156 (86.3%)	
Black	10 (6.0%)	117 (8.7%)	
Other	1 (0.6%)	53 (4.0%)	
Unknown	2 (1.2%)	13 (1.0%)	
CDCC Score, n (%)			0.09
0	86 (51.8%)	603 (45.0%)	
1	43 (25.9%)	481 (35.9%)	
2	27 (16.3%)	187 (14.0%)	
3+	10 (6.0%)	68 (5.1%)	
Year of Diagnosis (median, IQR)	2015 (2012, 2016)	2013 (2010, 2016)	<0.01
Tumor Size (median, IQR), mm	7 (4, 8)	7 (5, 8)	0.69
Tumor Location, n (%)			0.07
RUL	60 (36.1%)	486 (36.3%)	
RML	8 (4.8%)	48 (3.6%)	
RLL	28 (16.9%)	228 (17.0%)	
LUL	53 (31.9%)	350 (26.1%)	
LLL	13 (7.8%)	201 (15.0%)	
Unknown	4 (2.4%)	26 (1.9%)	
Histology, n (%)			<0.01
Squamous cell carcinoma	57 (34.3%)	261 (19.5%)	
Adenocarcinoma	109 (65.7%)	1078 (80.5%)	
Insurance Status, n (%)			<0.01
Uninsured	3 (1.8%)	19 (1.4%)	
Private	18 (10.8%)	386 (28.8%)	
Medicaid	8 (4.8%)	84 (6.3%)	
Medicare	131 (78.9%)	827 (61.8%)	
Other	4 (2.4%)	11 (0.8%)	
Unknown	2 (1.2%)	12 (0.9%)	
Facility Type, n (%)			0.31
Community cancer program	7 (4.2%)	52 (3.9%)	
Comprehensive community	68 (41.0%)	471 (35.2%)	
Academic/research program	60 (36.1%)	582 (43.5%)	
Integrated network cancer program	31 (18.7%)	225 (16.8%)	
Unknown	0 (0.0%)	9 (0.7%)	
Median Household Income, n (%)			0.03
First quartile	30 (18.1%)	192 (14.3)	
Second quartile	30 (18.1%)	234 (17.5%)	
Third quartile	40 (24.1%)	257 (19.2%)	
Fourth quartile	44 (26.5%)	514 (38.4%)	
Unknown	22 (13.3%)	142 (10.6%)	

Abbreviations: IQR, interquartile range; CDCC, Charlson/Deyo comorbidity condition; RUL, right upper lobe; RML, right middle lobe; RLL, right lower lobe; LUL, left upper lobe; LLL, left lower lobe; SBRT, stereotactic body radiation therapy.

**Table 2:** Independent predictors of overall survival after cox proportional hazards adjustment for patients with stage IA NSCLC tumors ≤8 mm NSCLC.

Patient Characteristic	Hazard Ratio	95% CI	*p* Value
Age (per year)	1.03	1.02, 1.04	<0.01
Female vs. male	0.64	0.53, 0.78	<0.01
Race (ref = white)			
Black	0.95	0.66, 1.37	0.80
Other	0.80	0.39, 1.63	0.54
Year of diagnosis (per year)	0.94	0.91, 0.97	<0.01
Median household income (ref = quartile 1)			
Second quartile	0.92	0.68, 1.23	0.56
Third quartile	0.96	0.71, 1.29	0.79
Fourth quartile	0.64	0.48, 0.85	<0.01
Insurance type (ref = uninsured)			
Private	1.03	0.43, 2.46	0.94
Medicaid	1.17	0.46, 2.99	0.74
Medicare	1.10	0.46, 2.61	0.82
Other	0.53	0.13, 2.19	0.38
Distance from facility (per mile)	0.7 (0.4, 0.8)	0.7 (0.5, 0.8)	0.69
Facility type (ref = community cancer program)			
Comprehensive community clinic	1.48	0.90, 2.43	0.12
Academic/research program	1.22	0.74, 2.00	0.44
Integrated network cancer program	1.30	0.77, 2.19	0.32
CDCC score (ref = 0)			
1	1.28	1.03, 1.59	0.02
2	1.36	1.04, 1.78	0.03
3+	1.71	1.11, 2.63	0.02
Squamous cell carcinoma v adenocarcinoma	1.09	0.88, 1.35	0.43
Tumor size (per cm)	1.00	0.96, 1.05	0.95
Tumor location (ref = RUL)			<0.01
RML	0.86	0.50, 1.46	0.56
RLL	1.25	0.95, 1.63	0.11
LUL	1.11	0.88, 1.39	0.36
LLL	1.03	0.75, 1.40	0.84
Wedge Resection vs. SBRT	0.55	0.42, 0.73	<0.01

Abbreviations: CDCC, Charlson/Deyo comorbidity condition; RML, right middle lobe; RLL, right lower lobe; LUL, left upper lobe; LLL, left lower lobe; SBRT, stereotactic body radiation therapy.

**Table 3:** Propensity-matched preoperative and demographic characteristics for patients with stage IA NSCLC tumors ≤8 mm NSCLC, stratified by SBRT vs. wedge resection.

Patient Characteristic	SBRT (n = 130)	Wedge Resection (n = 130)	Absolute Standardized Difference (%)	*p* Value
Age (years), median (IQR)	71.5 (11)	71 (11)	6.7	0.64
Sex, n (%)				0.80
Male	56 (43.1%)	58 (44.6%)	3.1	
Female	74 (56.9%)	72 (55.4%)	3.1	
Race, n (%)				0.53
White	120 (92.3%)	123 (94.6%)	7.8	
Black	9 (6.9%)	7 (5.4%)	5.9	
Other	1 (0.8%)	0 (0%)	5.1	
CDCC Score, n (%)				0.69
0	67 (51.5%)	76 (58.5%)	14.5	
1	36 (27.7%)	33 (25.4%)	5.0	
2	20 (15.4%)	16 (12.3%)	8.6	
3+	7 (5.4%)	5 (3.8%)	6.6	
Year of Diagnosis (median, IQR)	2015 (2012, 2016)	2015 (2013, 2016)	11.5	0.28
Tumor Size (median, IQR), mm	7 (3)	7 (3)	1.6	0.35
Tumor Location, n (%)				0.76
RUL	50 (38.5%)	52 (49.0%)	3.2	
RML	6 (4.6%)	10 (7.7%)	15.1	
RLL	21 (16.2%)	23 (17.7%)	4.0	
LUL	42 (32.3%)	37 (28.5%)	8.4	
LLL	11 (8.5%)	8 (6.2%)	7.2	
Histology, n (%)				0.51
Squamous cell carcinoma	39 (30.0%)	44 (33.8%)	8.8	
Adenocarcinoma	91 (70.0%)	86 (66.2%)	8.8	
Insurance Status, n (%)				0.95
Uninsured	<10	<10	1.8	
Private	14 (10.8%)	15 (11.5%)	2.0	
Medicaid	<10	<10	6.7	
Medicare	103 (79.2%)	102 (78.5%)	1.7	
Other	<10	<10	6.0	
Facility Type, n (%)				0.78
Community cancer program	5 (3.8%)	3 (2.3%)	7.7	
Comprehensive community	51 (39.2%)	55 (42.3%)	6.3	
Academic/research program	52 (40.0%)	54 (41.5%)	3.1	
Integrated network cancer program	22 (16.9%)	18 (13.8%)	8.1	
Median Household Income, n (%)				0.50
First quartile	26 (20.0%)	30 (23.1%)	8.4	
Second quartile	27 (20.8%)	19 (14.6%)	15.3	
Third quartile	36 (27.7%)	33 (25.4%)	5.4	
Fourth quartile	41 (31.5%)	48 (36.9%)	11.2	

Abbreviations: IQR, interquartile range; CDCC, Charlson/Deyo comorbidity condition; RUL, right upper lobe; RML, right middle lobe; RLL, right lower lobe; LUL, left upper lobe; LLL, left lower lobe; SBRT, stereotactic body radiation therapy.

**Table 4:** Independent predictors of overall survival after cox proportional hazards adjustment for patients with stage IA NSCLC tumors ≤8 mm NSCLC who have no comorbidities.

Patient Characteristic	Hazard Ratio	95% CI	*p* Value
Age (per year)	1.03	1.00, 1.05	0.02
Female vs. male	0.59	0.43, 0.81	<0.01
Race (ref = white)			
Black	0.80	0.41, 1.56	0.52
Other	0.63	0.19, 2.07	0.45
Year of diagnosis (per year)	0.96	0.91, 1.01	0.08
Median household income (ref = quartile 1)			
Second quartile	0.72	0.42, 1.22	0.23
Third quartile	0.86	0.53, 1.39	0.54
Fourth quartile	0.52	0.32, 0.84	<0.01
Insurance type (ref = uninsured)			
Private	1.93	0.26, 14.60	0.52
Medicaid	1.94	0.22, 17.01	0.55
Medicare	2.70	0.35, 20.77	0.34
Other	1.88	0.16, 21.62	0.61
Distance from facility (per mile)	1.00	1.00, 1.00	0.62
Facility type (ref = community cancer program)			
Comprehensive community clinic	2.20	1.00, 4.86	0.05
Academic/research program	1.77	0.79, 3.94	0.17
Integrated network cancer program	2.12	0.93, 4.86	0.07
Squamous cell carcinoma vs. adenocarcinoma	1.15	0.81, 1.64	0.44
Tumor size (per mm)	1.03	0.96, 1.11	0.38
Tumor location (ref = RUL)			
RML	1.59	0.79, 3.19	0.19
RLL	1.18	0.76, 1.84	0.47
LUL	1.18	0.80, 1.72	0.40
LLL	1.33	0.82, 2.14	0.23
Wedge Resection vs. SBRT	0.51	0.34, 0.77	<0.01

Abbreviations: RUL, right upper lobe; RML, right middle lobe; RLL, right lower lobe; LUL, left upper lobe; LLL, left lower lobe; SBRT, stereotactic body radiation therapy.

**Table 5:** Propensity-matched preoperative and demographic characteristics for patients with stage IA NSCLC tumors ≤8 mm NSCLC who have no comorbidities, stratified by SBRT vs. wedge resection.

Patient Characteristic	SBRT (n = 65)	Wedge Resection (n = 65)	Absolute Standardized Difference (%)	*p* Value
Age (years), median (IQR)	74 (12)	75 (9)	16.1	0.31
Sex, n (%)				0.60
Male	27 (42.0%)	30 (46.0%)	9.6	
Female	38 (58.0%)	35 (54.0%)	9.6	
Race, n (%)				0.93
White	61 (94.0%)	60 (92.0%)	5.4	
Black	3 (5.0%)	4 (6.0%)	6.5	
Other	1 (2.0%)	1 (2.0%)	0.0	
Year of Diagnosis (median, IQR)	2014 (2012, 2016)	2015 (2012, 2016)	5.8	0.91
Tumor Size (median, IQR), mm	7 (3)	7 (2)	14.7	0.97
Tumor Location, n (%)				0.70
RUL	28 (43.0%)	34 (52.0%)	19.0	
RML	6 (9.0%)	4 (6.0%)	12.8	
RLL	9 (14.0%)	10 (15.0%)	4.1	
LUL	20 (31.0%)	15 (23.0%)	17.1	
LLL	2 (3.0%)	2 (3.0%)	0.0	
Histology, n (%)				0.85
Squamous cell carcinoma	21 (32.0%)	22 (34.0%)	3.6	
Adenocarcinoma	44 (68.0%)	43 (66.0%)	3.6	
Insurance Status, n (%)				0.99
Uninsured	<10	<10	3.4	
Private	<10	<10	3.7	
Medicaid	54 (83.0%)	55 (85.0%)	0.0	
Medicare	<10	<10	3.4	
Other				0.99
Facility Type, n (%)				0.67
Community cancer program	3 (5.0%)	3 (5.0%)	0.0	
Comprehensive community	24 (37.0%)	18 (28.0%)	19.6	
Academic/research program	27 (42.0%)	29 (45.0%)	6.2	
Integrated network cancer program	11 (17.0%)	15 (23.0%)	16.2	
Median Household Income, n (%)				0.82
First quartile	8 (12.0%)	10 (15.0%)	9.5	
Second quartile	14 (22.0%)	11 (17.0%)	11.6	
Third quartile	17 (26.0%)	20 (31.0%)	10.5	
Fourth quartile	26 (40.0%)	24 (37.0%)	6.3	

Abbreviations: IQR, interquartile range; RUL, right upper lobe; RML, right middle lobe; RLL, right lower lobe; LUL, left upper lobe; LLL, left lower lobe; SBRT, stereotactic body radiation therapy.

**Table 6:** Independent predictors of overall survival after cox proportional hazards adjustment for patients with stage IA NSCLC tumors ≤8 mm NSCLC where SBRT arm received ≥50 Gy.

Patient Characteristic	Hazard Ratio	95% CI	*p* Value
Age (per year)	1.03	1.02, 1.04	<0.01
Female vs. male	0.64	0.53, 0.78	<0.01
Race (ref = white)			
Black	0.95	0.66, 1.37	0.80
Other	0.80	0.39, 1.63	0.54
Year of diagnosis (per year)	0.94	0.91, 0.97	<0.01
CDCC score (ref = 0)			
1	1.28	1.03, 1.58	0.02
2	1.36	1.04, 1.78	0.03
3+	1.71	1.11, 2.64	0.02
Median household income (ref = quartile 1)			
Second quartile	0.92	0.68, 1.23	0.56
Third quartile	0.96	0.71, 1.29	0.79
Fourth quartile	0.64	0.48, 0.85	<0.01
Insurance type (ref = uninsured)			
Private	1.03	0.43, 2.46	0.94
Medicaid	1.17	0.46, 2.99	0.74
Medicare	1.10	0.46, 2.61	0.83
Other	0.53	0.13, 2.19	0.38
Distance from facility (per mile)	1.00	1.00, 1.00	0.30
Facility type (ref = community cancer program)			
Comprehensive community clinic	1.48	0.90, 2.43	0.12
Academic/research program	1.22	0.74, 2.01	0.44
Integrated network cancer program	1.30	0.77, 2.19	0.32
Squamous cell carcinoma vs. adenocarcinoma	1.09	0.88, 1.35	0.43
Tumor size (per mm)	1.00	0.96, 1.05	0.95
Tumor location (ref = RUL)			
RML	0.86	0.50, 1.46	0.57
RLL	1.25	0.95, 1.64	0.11
LUL	1.11	0.88, 1.40	0.36
LLL	1.03	0.76, 1.41	0.84
Wedge Resection vs. SBRT	0.55	0.42, 0.73	<0.01

Abbreviations: CDCC, Charlson/Deyo comorbidity condition; RUL, right upper lobe; RML, right middle lobe; RLL, right lower lobe; LUL, left upper lobe; LLL, left lower lobe; SBRT, stereotactic body radiation therapy.

## Data Availability

The dataset, National Cancer Database, is publicly available through the American College of Surgeons https://www.facs.org/quality-programs/cancer-programs/national-cancer-database/ (accessed on 4 February 2021). Specific data used for this study are available from the authors upon reasonable request.
